# Introduction of Plasmid to the Murine Gut via Consumption of an *Escherichia coli* Carrier and Examining the Impact of Bacterial Dosing and Antibiotics on Persistence

**DOI:** 10.1007/s40883-022-00248-z

**Published:** 2022-04-14

**Authors:** LeNaiya Kydd, Fawaz Alalhareth, Ana Mendez, Maryann Hohn, Ami Radunskaya, Hristo Kojouharov, Justyn Jaworski

**Affiliations:** 1 Department of Bioengineering, The University of Texas at Arlington, Arlington, TX 76010 USA; 2 Department of Mathematics, The University of Texas at Arlington, Arlington, TX 76010 USA; 3 Department of Mathematics, Pomona College, Claremont, CA 91711 USA

**Keywords:** Microbiome, *Escherichia coli*, Antibiotic, Gene transfer

## Abstract

**Purpose:**

We examine the impacts of dosing strategies of plasmids on bacterial communities in the murine gut by measuring the quantity of plasmids in mouse feces.

**Methods:**

We fed mice carrier bacteria, *E. coli*, that contain plasmids with both a reporter gene and an antibiotic resistant gene. We varied the quantity of the plasmid-carrying bacteria and the length of time the mice consumed the bacteria. We also pretreated the gut with broad-spectrum antibiotics and used continuous antibiotic treatment to investigate selection pressure. We collected bacteria from fecal pellets to quantify the number of plasmid-carrying bacteria via plate assay.

**Results:**

Dosing regimens with plasmid-carrying bacteria resulted in a significantly increased duration of persistence of the plasmid within the gut when supplemented continuously with kanamycin during as well as after completion of bacterial dosing. The carrier bacteria concentration influenced the short-term abundance of carrier bacteria.

**Conclusion:**

We evaluated the persistence of plasmid-carrying bacteria in the murine gut over time using varying dosage strategies. In future work, we will study how bacterial diversity in the gut impacts the degree of plasmid transfer and the prevalence of plasmid-carrying bacteria over time.

**Lay Summary:**

Observing how plasmids persist within the gut can help us understand how newly introduced genes, including antibiotic resistance, are transmitted within the gut microbiome. In our experiments, mice were given bacteria containing a genetically engineered plasmid and were examined for the persistence of the plasmid in the gut. We found long-term persistence of the plasmid in the gut when administering antibiotics during and following dosing of the mice with bacteria carrying the plasmid. The use of higher concentrations of carrier bacteria influenced the short-term abundance of the plasmid-carrying bacteria in the gut.

**Description of Future Works:**

Building on evidence from these initial studies that persistence of plasmids within the gut can be regulated by the dosage strategy, we will explore future studies and models of gene uptake in the context of spatial and taxonomic control and further determine if dosing strategies alter the compositional diversity of the gut microbiome.

**Graphical abstract:**

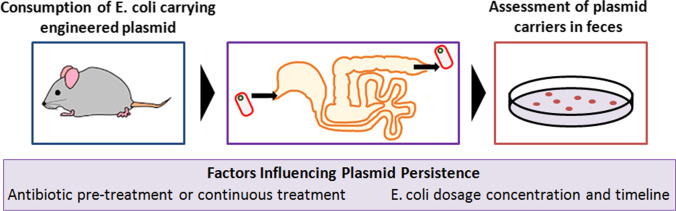

## Introduction

In the mammalian gut, dynamics of the environment, including continuous secretion of mucin, limit direct colonization of the underlying epithelium but permit the existence of high-density microbial communities [1, 2]. The diversity of species within and across hosts is believed to be spatially structured [3], as it is well known that the high bacterial densities presented in the mammalian gut, up to 10^3^ CFU/mL for the stomach and duodenum, 10^8^ CFU/mL for the jejunum and ileum, and 10^12^ CFU/g for the colon, are conducive to gene transfer [4]. Gene transfer is a frequent event among bacteria [5, 6]. Understanding these dynamics is important in order to recognize the impact that gene transfer events have on the ability to break down food and drugs, the emergence of antibiotic resistance, and the communication with the host immune system [2, 3, 7–9]. The introduction of new genetic elements to gut bacteria has been proposed as a way of modifying their metabolic capabilities. To make this possible, we need to understand the transmission and persistence of mobile genetic constructs within the gut. In this work, we examine the introduction of a plasmid to the murine gut to measure its ability to persist within the microbial community of the gastrointestinal tract. We study the effect of dosage duration of carrier bacteria, dosage concentration of carrier bacteria, usage of antibiotic pre-treatment, and continuous supplementation of antibiotic for selection pressure on gut bacteria. By developing a mathematical model calibrated to experimental data, we will be able to efficiently test multiple dosing strategies and conditions.

Past approaches to studying gene transfer in the gut include culture-based techniques and high-throughput fluorescent systems in which lab animals are fed bacteria containing antibiotic resistant or fluorescent reporter genes to isolate transconjugants by selection or screening, respectively [10, 11]. A recent study used fluorescence-activated cell sorting (FACS) of the bacterial recipients of plasmids in a microbial community extracted from soil showing that this heterogeneous community provides a hot-spot for gene acquisition from phylogenetically distant groups, as introduced plasmids were found to be hosted by very diverse bacteria [12]. Data have supported that the host range of plasmid recipients is carrier-dependent [13, 14], and it has also been shown that plasmid fitness effects or costs to the host can differ between bacteria, ultimately impacting plasmid persistence in bacterial communities [15–18]. A recent review highlighted examples of engineered bacteria that were clinically tested to afford gain-of-function to the gut microbiome. A particular emphasis has been placed on conveying the need for more rigorous testing and modeling for assessing stability and safety [19]. Approaches for quantifying the stability of engineered *E. coli* have shown that the mutation rate and overall fitness of a gene construct when not under selective pressure will be affected by the number of repeated sequences and the gene expression [20].

To discover the effect of dosing schedule, dosing concentration, and pre-treatment on the persistence of the plasmid-carrying bacteria in the gut, we study the persistence of plasmids within *E. coli* that carry a set of antibiotic selectable and fluorescently screenable genetic markers. We hypothesize that the persistence of the plasmid depends on the conditions under which the plasmid-carrying *E. coli* were introduced to the gut, including variations in antibiotic pre-treatment, continuous antibiotic supplementation, and duration and concentration of the dosage of the plasmid-carrying *E. coli*. To test this, we performed a series of plate-based culture assays to quantify the amount of plasmid-carrying bacteria within the fecal samples of mice that received doses of plasmid-carrying *E. coli*. As described, we have identified that the persistence of an introduced plasmid can be extended significantly by providing continuous antibiotic selection pressure for carrier bacteria to retain the plasmid carrying the corresponding antibiotic resistance gene. A two-dimensional mathematical model with seven parameters was fit to the experimental setting allowing us to make qualitative predictions in virtual conditions and dosing regimens. While this study is limited to examining only a single form of carrier bacteria and plasmid, we believe this initial work will serve as a step toward future studies with the goal of predicting and potentially controlling the duration of persistence for plasmids introduced to the gut.

## Methods

### Carrier Bacteria Preparation, Delivery to Mice, and Quantification from Fecal Pellets

**Fig. 1:**
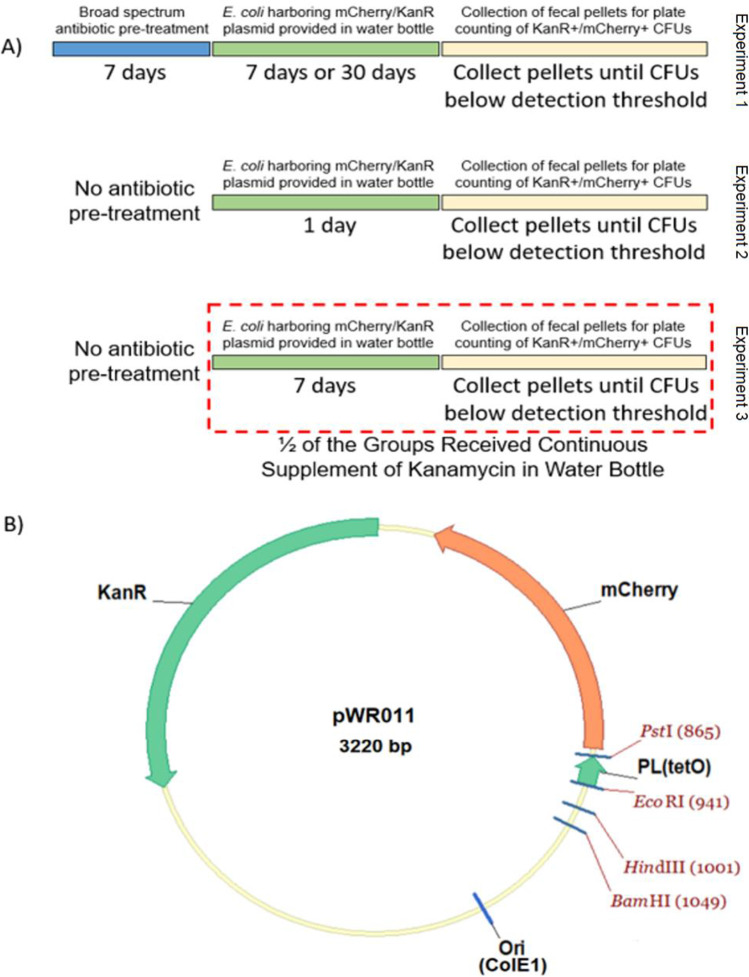
**A** Overview of the workflow for mouse studies (experiments 1 to 3) in which *E. coli* carrying the pWR011 plasmid is provided to mice at distinct dosage concentrations, durations, and the controlled use of antibiotics. **B** Plasmid map of pWR011

To begin examining the persistence of a plasmid within the gut microbiota, we selected a plasmid pWR011 containing a kanamycin resistance cassette, a ColE1 high copy origin of replication, and a red fluorescent mCherry gene under the control of a PL(tetO) promoter (see Fig. 1B for a schematic of the plasmid construct). This plasmid was transformed into chemically competent *E. coli* of the human-derived strain Nissle 1917 that then served as the carrier bacteria for the murine studies. Mice did not experience adverse events resulting from the *E. coli* consumption studies, and all studies were carried out in accordance with our institution’s IACUC approval. Unless otherwise specified, mice were housed at 4 mice per cage, and their average liquid consumption from their water bottles was recorded. We controlled water bottle content such as the amount of water, antibiotic, carrier *E. coli*, and/or any mixtures, depending on the experimental conditions. The presence of the reporter gene and selectable resistance allowed the bacteria which received and expressed the plasmid to be easily discerned by plating onto selective media. To determine the number of pWR011-carrying bacteria per fecal pellet, we gathered four fecal pellet samples per condition, re-suspended them in 0.5 mL of sterile PBS, and after making serial dilutions, we plated 5uL of each dilution onto LB-agar (Lennox media formulation) petri dishes containing kanamycin (50ug/mL). Since only 1/100th of the sample was used during plating, the CFU detection threshold for these studies was a minimum of 100 CFU per pellet for detection. For each mouse condition at each timepoint, we replicated 5 plates from the same dilution for plating. Using these plate-based cultures, we determined the number of cultural plasmid-carrying bacteria within each fecal pellet sample. The antibiotic and reporter genes allowed for simple quantification of the number of CFUs per pellet since those bacteria form colonies expressing mCherry in the presence of 50ug/mL kanamycin. We performed three separate experiments as depicted in Fig. 1A.

### Comparison of Length of Carrier Dosage and Antibiotic Pre-treatment on Persistence (Experiment 1)

In experiment one, we used 16 Swiss-Webster mice (4 weeks of age from Charles River Labs, Wilmington, MA) to examine the effects of antibiotic pre-treatment and length of dosing. We separated them into 4 groups: no antibiotic pre-treatment + 1 week of carrier bacteria; no antibiotic pre-treatment + 1 month of carrier bacteria; antibiotic pre-treatment + 1 week of carrier bacteria; and antibiotic pre-treatment + 1 month of carrier bacteria. For conditions in which we provided antibiotic pre-treatment, we filled water bottles with an aqueous solution of 1 g/L ampicillin, 1 g/L neomycin sulfate, 1 g/L metronidazole, 0.5 g/L vancomycin, and 5% sucrose. Then, we gave these water bottles to the mice for 7 days prior to dosing with carrier bacteria. We administered the carrier bacteria treatment by replacing the liquid in their water bottles with an aqueous solution containing 106 CFU/mL of carrier *E. coli* possessing the pWR011 plasmid. The mice drank the aqueous solution ad lib, and we calculated the average amount of bacteria consumed per day per mouse by weighing the individual water bottles to find the volume of liquid consumed. We collected fecal pellets to quantify the number of colony-forming units (CFU) of pWR011-carrying bacteria using the methods outlined above.

### Comparison of Carrier Concentration of Dosage on Persistence (Experiment 2)

In experiment two, we separated 24 Swiss-Webster mice into 3 groups of 8 (4 male and 4 female). Without any antibiotic pre-treatment, we gave the mice water bottles containing an aqueous solution of low dosage concentration (5 × 10^6^ CFU/mL), medium dosage concentration (5 × 10^7^ CFU/mL), or high dosage concentration (5 × 10^8^ CFU/mL) of pWR011 carrier bacteria. The mice consumed the aqueous solution ad lib for 24 h, and we calculated the average amount of bacteria consumed per day per mouse by weighing the individual water bottles and finding the volume of liquid consumed. We assessed the number of CFU of pWR011-carrying bacteria in collected fecal pellets by the plating procedure described above.

### Comparison of Selection Pressure with Continuous Antibiotic Treatment on Persistence (Experiment 3)

In experiment three, we separated 24 Swiss-Webster mice into 4 groups of 6 mice per group (3 male and 3 female). Without any antibiotic pre-treatment, the mice were provided over a period of 7 days with a water bottle containing pWR011 carrier *E. coli* under one of the following four conditions: (#1) medium dosage concentration (5 × 10^7^ CFU/mL of *E. coli*), (#2) high dosage concentration (5 × 10^8^ CFU/mL of *E. coli*), (#3) medium dosage concentration (5 × 10^7^ CFU/mL of *E. coli*) with 25 µg/mL kanamycin, or (#4) high dosage concentration (5 × 10^8^ CFU/mL *E. coli*) with 25 µg/mL kanamycin. The presence of the kanamycin supplement served the purpose of providing selection pressure as the pWR011 plasmid contains the kanamycin resistance cassette (KanR). After 7 days, we replaced the water bottles with fresh water for conditions #1 and #2 listed above. In contrast, we replaced the water bottles with water containing 25 µg/mL kanamycin for conditions #3 and #4 to provide selection pressure. We determined the number of CFU of pWR011-carrying bacteria in collected fecal pellets as described above. Again, we allowed the mice to drink the contents of the water bottle ad lib, and we calculated the average amount of liquid consumed per day per mouse by weighing the individual water bottles.

## Results

**Fig. 2:**
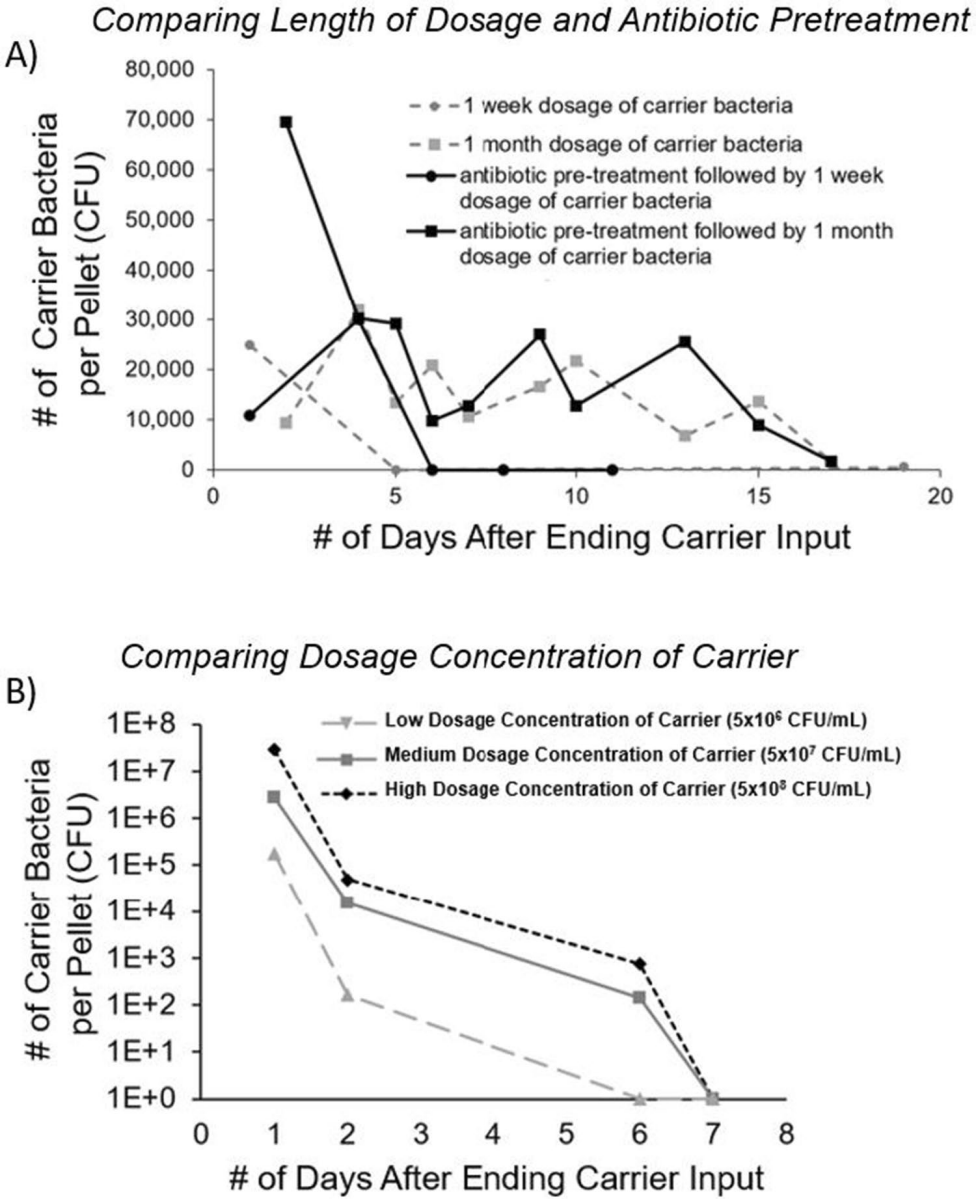
**A** Results from experiment 1 showing the amount of pWR011 plasmid-carrying bacteria that were found in the fecal pellets of mice after consuming the carrier bacteria for either 1 week or 1 month under the conditions of 1-week broad-spectrum antibiotic pre-treatment or no pre-treatment. **B** Results from experiment 2 showing the amount of pWR011 plasmid-carrying bacteria in the fecal pellets after providing mice with a water bottle over a single 24-h period containing a low dosage concentration of 5 × 10^6^ CFU/mL, a medium dosage concentration of 5 × 10^7^ CFU/mL, or a high dosage concentration of 5 × 10^8^ CFU/mL of the pWR011 carrier bacteria

In order to examine the persistence of pWR011 plasmid carrier bacteria in the murine gut, we analyzed the number of colony-forming units (CFUs) from fecal pellets selected on kanamycin-containing media. Although a typical fecal pellet was approximately 0.02 g, there were visible variations in the pellet size across samples. This size variation was not recorded in this study, but it is expected to be a factor in the noticeable fluctuations in the number of carrier bacteria per pellet collected over time. The results of the fecal pellets’ bacterial content reveal the number of CFUs of bacteria in the fecal pellet that possess the pWR011 plasmid for each of the distinct dosage conditions as outlined below. In Fig. 2A, the data reveal two distinct conditions: either (1) 1 week of pre-treatment with broad-spectrum antibiotics followed by 1 week of consumption of bacteria via water bottles containing 10^6^ CFU/mL of plasmid-carrying *E. coli* or (2) 1 week of consumption of bacteria from water bottles with 10^6^ CFU/mL of plasmid-carrying *E. coli* but no antibiotic pre-treatment. Having provided 10^6^ CFU/mL of carrier bacteria and recording the amount of liquid consumed to range between 4 and 9 mL per mouse per day, we determined that 4 × 10^6^ to 9 × 10^6^ bacteria were consumed per mouse per day. After 1 week of providing the carrier bacteria to the mice via water bottles, the bottles were replaced with fresh water without bacteria, and the fecal pellets were collected beginning the day after receiving fresh water. After providing carrier bacteria at 10^6^ CFU/mL for 1 week, we measured the persistence of plasmid-carrying bacteria in the fecal pellets at a level above 10^4^ CFUs per pellet on days 1 and 4 for the case of antibiotic pre-treatment but on only day 1 for the case of no antibiotic pre-treatment.

To determine if a longer dosage period for providing the plasmid-carrying bacteria to the mice would result in a change in the observed persistence of the plasmid, we also examined (as seen in Fig. 2A) the presence of carrier bacteria in the fecal pellets of the mice that received 1 month of the pWR011 plasmid-carrying *E. coli*. In looking at these longer dosing timescales, we can see that the number of plasmid-carrying bacteria in the fecal pellets remained above the level of 10^4^ of CFU/pellet for 15 days in both the case of mice having undergone antibiotic pre-treatment and mice having no antibiotic pre-treatment prior to this 1 month (30 days) of ad lib consumption of plasmid-carrying *E. coli* to the mice at 10^6^ CFU/mL. In comparison to our 1-week dosing study, it appears that this longer dosage time of 1 month for the plasmid carrier *E. coli* could result in extended persistence of 15 days as compared to persistence of only 4 days if dosing with carrier bacteria for 1 week.

In experiment 2 (Fig. 2B), we gave mice without antibiotic pre-treatment three different dosage concentrations of pWR011 plasmid carrier *E. coli* bacteria in their water bottles for a single 24-h period. The concentrations spanning three orders of magnitude were a low dosage concentration of 5 × 10^6^ CFU/mL, a medium dosage concentration of 5 × 10^7^ CFU/mL, or a high dosage concentration of 5 × 10^8^ CFU/mL of the pWR011 carrier bacteria. The data in Fig. 2B affirms the expectation that a higher number of carrier bacteria initially appeared in the pellet for mice, providing the higher concentration dosing of carrier bacteria. These results indicate that the higher dosage concentration of consumed carrier bacteria provided an increased timeframe of plasmid persistence. Specifically, 2 days of plasmid carrier persistence was observed after a single day dosage of low concentration carrier CFU, while 6 days of plasmid carrier persistence was seen after a single day dosage of either medium or high concentration of carrier.

**Fig. 3:**
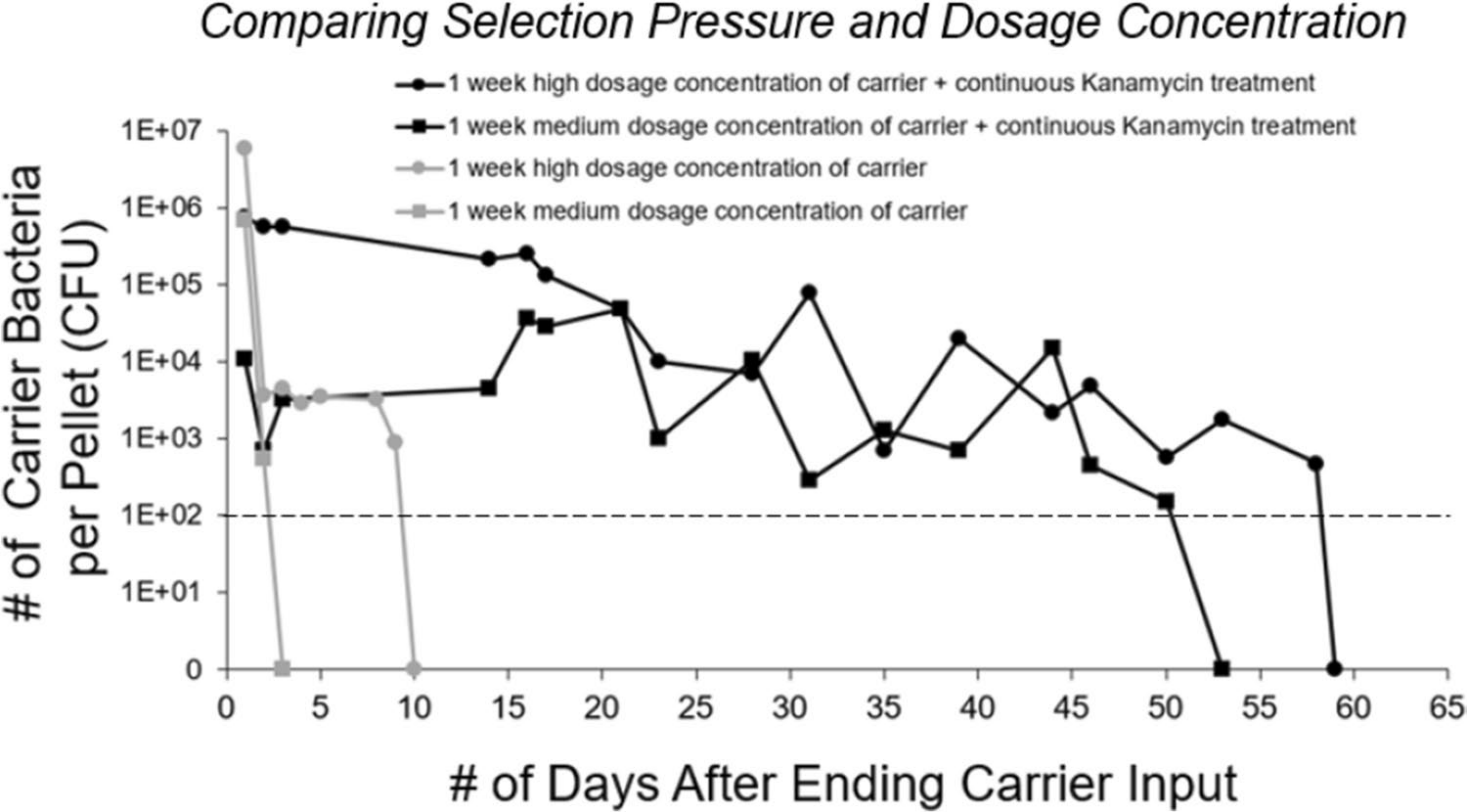
Results from experiment 3 show the amount of pWR011 plasmid-carrying bacteria in the fecal pellets after providing mice with water bottles containing a medium dose concentration of 5 × 10^7^ CFU/mL of carrier bacteria or a high dosage concentration of 5 × 10^8^ CFU/mL with either the addition of 25 µg/mL kanamycin selection pressure or without kanamycin. The dashed horizontal line at 100 CFU/pellet represents the limit of detection

To examine if increased persistence could be achieved by providing a selection pressure for the bacteria to carry the plasmid, we provided half of the mice in experiment 3 with a continuous supply of kanamycin in their water bottles along with a 1-week continuous dose of either medium or high dosage concentration of pWR011-carrying *E. coli*. After 1 week of bacterial dosing, those mice receiving simultaneous kanamycin continued to receive the kanamycin supplement alone in their water bottles. Because the pWR011 plasmid imparts kanamycin resistance, the selective advantage for carrying the plasmid was expected to enhance its persistence in the gut, as seen in Fig. 3. The data demonstrated that after 1 week of providing carrier bacteria to the mice at either the high or medium dosage concentration, a significant increase in the persistence of plasmid-carrying bacteria could be seen for the mice that were also provided a continuous supply of kanamycin as a selection pressure. Without the continuous supply of kanamycin, the number of carrier bacteria remained detectable for 3 to 10 days, respectively, for 1 week of medium or high dosage concentration of carrier bacteria. In contrast, at those same dosages and provided a continuous supply of kanamycin, the carrier bacteria population remained detectable (above our 100 CFU detection limit) for 50 to 55 days (Fig. 3). Directly comparing the two conditions, we observed a statistically significant increase in persistence when applying selection pressure (48 ± 14 days) as compared to no selection pressure (6 ± 4 days) (*P*-value = 0.007 using a two-tailed *t*-test).

Using the data from experiment 3, we developed a model for mice given antibiotics consistently or without antibiotics using a two-population logistic growth model based on the introduction of a carrier species harboring a transferrable plasmid that confers antibiotic resistance as well as a second population that does not possess the antibiotic resistance but that may become resistant upon receiving the plasmid. The following are the model equations for each population:

$$\begin{array}{cc}\frac{d{B}_{1}}{dt}={\alpha }_{1}{B}_{1}\left(1-\frac{{B}_{1}+{B}_{2}}{K}\right)-\gamma {B}_{1}+{\eta B}_{2}-D{B}_{1}& \frac{d{B}_{2}}{dt}={\alpha }_{2}{B}_{2}\left(1-\frac{{B}_{1}+{B}_{2}}{K}\right)\end{array}+\gamma {B}_{1}-{\eta B}_{2}-D{B}_{2}-\lambda {B}_{2}$$

**Fig. 4:**
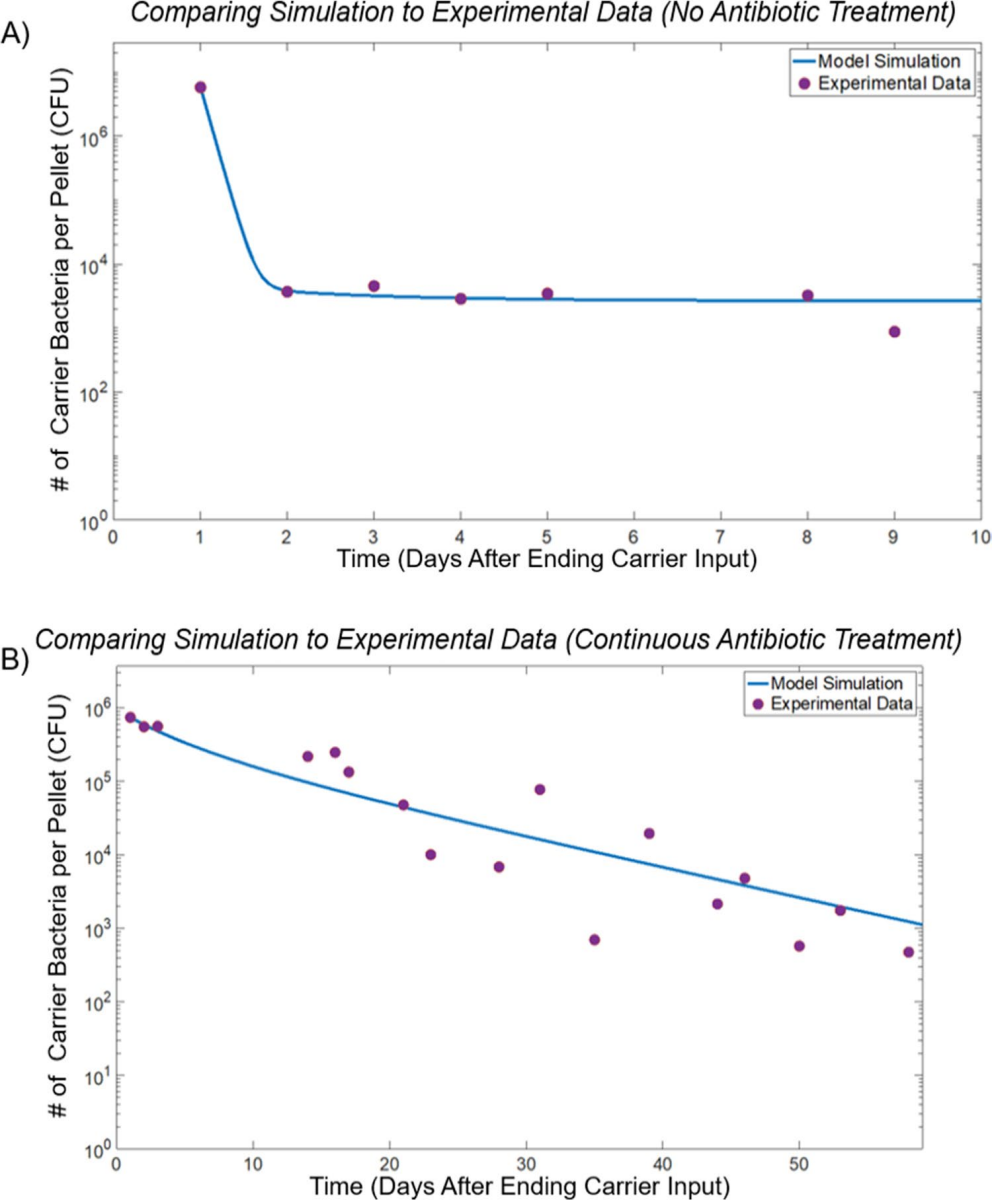
Preliminary models of persistence of bacteria introduced to the murine gut based on two-population logistic growth for high dosage concentration of plasmid-carrying bacteria **A** without continuous antibiotic treatment and **B** with continuous antibiotic treatment as selection pressure using the parameters of *D* = 3.25, *K* = 200,000, *α*_1_ = 3.0, *α*_2_ = 3.7848, *η* = 0.4973, and *γ* = 10 for the model without antibiotic and *D* = 3.25, *K* = 200,000, *α*_1_ = 3.2504, *α*_2_ = 3.2925, *η* = 0.01, *γ* = 0.1, and *λ* = 0.3 for the model with antibiotic. The simulations from the model are presented as blue lines, and the purple circles are the high dosage concentration condition data points from experiment 3 without and with continuous kanamycin supplementation, respectively

Here, *B*_1_ is the plasmid carrier bacteria, *B*_2_ is the non-carrier bacteria, *K* is the carrying capacity of the environment, *D* is the dilution rate of bacteria due to defecation, *α*_1_ and *α*_2_ are the intrinsic growth rate constants of *B*_1_ and *B*_2_, *η* is the rate of plasmid transfer from carrier to non-carrier, *γ* is the rate of plasmid loss, and *λ* is the antibiotic-induced death rate. In Fig. 4, the model is fit to our data of the high dosage concentration of carrier bacteria with the units of the number of bacteria present per fecal pellet, and time is measured in days. The fits were carried out using MATLAB’s built-in constrained optimization routine “fmincon” with the “multistart” option. The parameters were constrained to be in a prescribed interval, and the fitting routine started at multiple points in this constrained parameter space in order to distinguish between local minima. The parameters *α*_1_, *α*_2_, *η*, and *γ* were first fitted to data from the experiment without antibiotics (Fig. 4A), and then using these values of *η* and *γ*, the growth and death parameters (*α*_1_, *α*_2_, and *λ*) were fitted to the experimental data for the antibiotic model (Fig. 4B). Numerical simulations were run using the built-in ordinary differential equations solver ode45 in MATLAB.

## Discussion

Our observations that mice provided a 1-week dosage of carrier *E. coli* show plasmid carrier persistence for 4 days are consistent with prior work showing that *E. coli* K12 can survive in the human intestine for nearly a week after a single dose of > 10^4^ CFU [21]. In another example from the literature in which *E. coli* were genetically engineered to possess a chromosomally integrated lambda-epigenetic switch as a memory element for detected chemical cues, it was shown that 8 days after the final dosage of the K12-based *E. coli* strain, the amount of remaining engineered bacteria in the fecal pellet was less than 1 CFU per mg, where a typical fecal pellet may be approximately 20 mg.Thus, the engineered bacteria were almost completely outcompeted by the natural gut flora, while a higher amount (of ~ 1000 CFU per mg) was identified on day 8 for a similarly engineered NGF-1 strain [22]. We found that increasing the single day dosage amounts of carrier bacteria produced noticeably higher abundances of plasmid carrier in the initial days after completing the dosing regimen. In looking at the longer dosing timelines, we saw an increased duration of the persistence of the plasmid within the gut. This could be due to the longer duration course of consumed bacteria harboring the plasmid providing more opportunities for transfer of the plasmid to the existing commensal gut bacteria, or alternatively, it could be due to the longer dosing allowing the consumed bacteria a better chance of colonizing the gut.

It has been shown that the stable introduction of exogenous bacterial strains for conditioning to the gut may benefit from the use of antibiotic pre-treatment to reduce competition with resident gut bacteria [23]. Our comparison between broad-spectrum antibiotic pre-treated mice and untreated mice did not reveal differences in persistence, but a higher carrier bacteria abundance was observed for antibiotic pre-treated mice immediately after the carrier *E. coli* dosage period, which may be reasonably attributed to increased survival of the introduced *E. coli*. There are several reports in the literature of the effects of competition from resident bacteria on colonization [24–26]; however, we cannot say definitively from this study that antibiotic pre-treatment reduces commensal bacteria to increase carrier survival. For example, in our 1-month and 1-week study shown in Fig. 2A, we observed no significant effects of antibiotic pre-treatment on the persistence. We did, in contrast, find a significant increase in the persistence of the pWR011-carrying bacteria in the fecal pellets when providing selection pressure, as seen in Fig. 3, comparing mice having consumed bacteria with and without the continuous supplement of kanamycin.

This work represents our first attempts to examine the persistence of plasmid within carrier bacteria. Further work using refined approaches is needed to uncover possible underestimation of the number of plasmid-carrying bacteria. Not all transconjugants such as resident bacteria that have received the plasmid are capable of being cultured on the selected media used here. Our preliminary mathematical model predicts that, when an antibiotic is administered persistently over a period of time, the growth rate of the non-carrier bacteria decreases, while the growth rate of the carrier bacteria increases slightly. This model qualitatively describes the selection pressure due to continuous kanamycin supplementation. We will build on this model in future work, where we will incorporate more details of the processes associated with the transfer and maintenance of plasmids. We will include aspects of plasmid stability and an additional population not capable of carrying the plasmid to better represent the physical system. Additionally, we will carry out a sensitivity analysis that will illustrate which parameters are most influential in the model.

## Conclusion

Through this study, we provide an assessment of the plasmid carrier’s persistence in the murine gut. We revealed the effect of duration in plasmid carrier dosing as well as selection pressure by continuous antibiotic treatment on persistence. While serving as a starting point for more sophisticated studies of gene transfer, this work can provide a foundation for selecting plasmid carrier dosing conditions when examining the dynamics of genetic constructs through the murine gut. We have also provided a very simple preliminary model, which displays a qualitative fit; however, we aim to explore more advanced models that provide accurate approximations for more complex processes. Future work in discerning between carrier and recipient bacteria among the carrier bacteria will also help to improve our understanding of the degree of gene transfer, and examining compositional changes in the bacterial diversity of the recipients over time will provide valuable information on rates of carrier-recipient transfer. Finally, we anticipate that continued work on iteratively improving these models will help to direct future experiments and, ultimately, lead to a better understanding of the persistence of mobile genetic elements in the gut.

## Data Availability

All data collected has been provided within the manuscript.

## Declarations

### Additional Declarations for Articles in Life Science Journals that Report the Results of Studies Involving Humans and/or Animals

The studies have been approved by the Institutional Animal Care and Use Committee (IACUC) at UT Arlington where we conducted our study (Animal Care Assurance Number: A3169-01).
